# Investigation of Possible Positive Effects of Arbutin Application in Experimental Colitis Model

**DOI:** 10.5152/tjg.2024.23205

**Published:** 2024-07-01

**Authors:** Merve Bıyıklı Alemdar, Ferhat Şirinyıldız, Adil Coşkun, İbrahim Meteoğlu, İsmail Taşkıran, Altay Kandemir, Mehmet Hadi Yaşa

**Affiliations:** 1 Department of Internal Medicine, Adnan Menderes University Faculty of Medicine, Aydın, Türkiye; 2 Department of Gastroenterology, Adnan Menderes University Faculty of Medicine, Aydın, Türkiye; 3 Department of Physiology, Adnan Menderes University Faculty of Medicine, Aydın, Türkiye; 4 Department of Pathology, Adnan Menderes University Faculty of Medicine, Aydın, Türkiye

**Keywords:** Arbutin, experimental animal model, mesalazine, ulcerative colitis

## Abstract

**Background/Aims:**

This study aimed to investigate the possible positive effects of arbutin in a trinitrobenzene sulfonic acid (TNBS)-induced experimental colitis model, to compare it with mesalazine, which is used in treating inflammatory bowel disease and to observe the effect of its concomitant use.

**Materials and Methods:**

Forty Wistar albino species male rats were randomized into 5 groups as control, colitis, colitis + arbutin (Arb), colitis + mesalazine (Mes), and colitis + mesalazine + arbutin (M+A). Proinflammatory cytokines [interleukin (IL)-6, IL-1β, tumor necrosis factor alpha (TNF-α)] and oxidant/antioxidant parameters [malondialdehyde (MDA), superoxide dismutase inhibition (SOD) inhibition, myeloperoxidase (MPO), and catalase, glutathione peroxidase (GPx)] were processed from the samples. Histopathological evaluation evaluated goblet cell reduction, cellular infiltration, and mucosal loss.

**Results:**

When the treatment groups and the TNBS group were compared, statistical significance was achieved in MDA, MPO, SOD inhibition, GPx values, IL-6, IL-1β and TNF-α levels. Histopathological evaluation revealed a statistically significant decrease in the mucosal loss value in the group where mesalazine and arbutin were used together compared to the TNBS group.

**Conclusion:**

Our study’s results elaborated that using arbutin alone or in combination with mesalazine produced positive effects in colitis-induced rats.

**Main Points**

- Arbutin has anti-inflammatory and antioxidant effects.
- Arbutin has a protective and therapeutic effect on colitis.
- Colon structure deteriorated with trinitrobenzene sulfonic acid is healed by arbutin administration.

## Introduction

Ulcerative colitis (UC) is an inflammatory disease that affects the gastrointestinal tract becomes chronic and progresses with relapses. Its etiology and pathogenesis still need to be fully elucidated. However, in recent years, significant progress has been made in revealing the pathogenesis of the disease. Genetics, immunological factors, intestinal flora, and environmental characteristics are thought to play a role in the etiology. Treatment of UC is non-specific. In general, it is based on agents with anti-inflammatory activity.^1,2^ The main goals of treatment in UC are to achieve remission, increase the patient’s quality of life, and prevent complications that may develop.^3^ Experimental colitis models, particularly 2,4,6 trinitrobenzene sulfonic acid (TNBS), have been utilized in various experimental animal models; a single fast, reliable, and reproducible method is essential for exhibiting pharmacological approaches to UC.^4^

Experimental and clinical studies show that triggering oxidative stress advances the development of IBD. There is a correlation between the increase of reactive oxygen species in UC and the degree of inflammation. Oxidative stress damages the gastrointestinal mucosa and bacterial invasion, stimulating the immune response and initiating IBD.^5,6^

Anti-inflammatory agents and antioxidant defense mechanisms positively affect the course of IBD by preventing the initiation of lipid peroxidation, forming free radicals, and increasing cytokines.^7^ Mesalazine, 5-aminosalicylic acid (5-ASA), is the standard for treating uncomplicated, mild to moderate UC. Meta-analyses of randomized controlled trials have shown the superiority of mesalazine over placebo and rectal steroids in both remission and maintenance therapy in the treatment of UC.

Mesalazine can be administered rectally through suppositories, foams, or enemas.^8,9^ It is a β-glucoside derived from arbutin hydroquinone (HQ; 1,4-dihydroxybenzene), which is obtained from the leaves of various plants, such as blueberries. The leaves of these plants have traditionally been used for many years by the natives of China and the American continent as folk remedies, especially for wound healing and treating urinary tract infections.^10-15^ Arbutin has been shown to have long-term free radical removal properties and protective properties from oxidative stress by inhibiting lipid peroxidation.^10,13,16^ Studies on arbutin have shown that it increases the anti-inflammatory effects of corticosteroids and indomethacin. In addition, arbutin reduces free radical formation in neutrophils and decreases enzyme release from neutrophils in experimental arthritis and in vitro studies.^17^ Experimental studies on intestinal epithelial cells have shown that arbutin increases cell viability.^18^

Although oxidative stress and antioxidant power level are in balance under physiological conditions, this balance is disrupted in the direction of oxidative damage in UC processes, disrupting the occurring inflammation process, increasing tissue damage, and prolonging the healing period. Therefore, biochemical analysis of oxidative damage and antioxidant biomarkers glutathione peroxidase (GPx), malondialdehyde (MDA), superoxide dismutase inhibition (SOD), myeloperoxidase (MPO), and catalase (CAT) levels, together with histopathological evaluation, reveals the positive efficacy of treatments applied against ulcerative colitis at the tissue level.

The study aimed to compare the positive effect of arbutin against induced UC with routine mesalazine therapy and combined therapy.

## Materials and Methods

This study was carried out in Aydın Adnan Menderes University Faculty of Medicine Experimental Animals Laboratory using a total of 40 Wistar albino species male rats with weights ranging from 250-400 g obtained from Aydın Adnan Menderes University Faculty of Medicine Experimental Animals Laboratory (ethical approval: HADYEK dated May 27, 2021, and 64583101/2021/073), biochemical analyses were performed in Aydın Adnan Menderes University Faculty of Medicine Physiology Laboratory, and histopathological analyzes were performed in Aydın Adnan Menderes University Faculty of Medicine Pathology Laboratory. The animals were allowed to be in pre-experimental cages in a controlled room with a 12-hour light–darkness cycle. They were fed with standard rat feed and allowed to drink water from the fad. After the environment was adapted, all the animals were starved 12 hours before the experiment, and the experiment was started (Table 1).

### Sham Control Group

Rats in this group were not exposed to colitis. Saline was administered intragastric and rectal by cannula.

### Colitis Group

The intestines of rats in this group and treatment groups were emptied, under ketamine (90 mg/kg) and xylazine (10 mg/kg) anesthesia, diluted in 0.8 mL saline of 25 mg of TNBS. The solution, prepared by dissolving in 37% ethanol, was given by entering 8 cm inside the anal orifice with the help of a polyethylene cannula.^19,20^ No therapeutic agents were administered to rats in the colitis group.

### Colitis + Mesalazine Group

The first 100 mg/kg mesalazine administration was performed intragastric 3 times, 6 hours after colitis first gavage was performed.

### Colitis + Arbutin Group

Arbutin 250 mg/kg was administered intragastrically 3 times, the first of which was 6 hours after colitis first gavage was performed.

### Colitis + Mesalazine + Arbutin Group

In the first one, 6 hours after the formation of colitis, 3 times 100 mg/kg mesalazine and 250 mg/kg arbutin administration were performed intragastric route.

Colitis was formed in all rats in the treatment groups, and no therapeutic agents were given before colitis was formed. Induction of experimental colitis was performed by applying the procedure in the colitis group. Seventy-two hours after colitis was formed, rats were sacrificed under ketamine xylazine anesthesia. At the end of the process, colon tissue was taken from the rats, and the experiment was terminated.

The tissues for biochemical analysis were quickly taken to a −80°C freezer and preserved until the analysis day. On the day of the analysis, homogenization of the tissues in phosphate buffer (1 : 20) was performed, and procedures were performed in accordance with the instructions inside the kits. Kits (BioVision, Milpitas, Calif, USA) was used to measure tissue GPx, MDA, SOD, MPO, and CAT levels. Tissue samples were followed by instructions with ScienceCell (Calif, USA) brand rat ELISA kits to measure interleukin-1 beta (IL-1β), interleukin-6 (IL-6), and tumor necrosis factor alpha (TNF-α) levels. All tests were analyzed with the help of the “Diagnostic Automation, Inc./ELx800TM” brand microplate reader.

The tissues were detected in 10% neutral buffered formalin and taken to routine tissue follow-up. After this procedure, 4 μm thick sections were prepared with rotary microtomes from tissue samples embedded in paraffin blocks. These sections were stained with hematoxylin eosin (HE) and evaluated at magnifications of ×4, ×10, ×20, and ×40 under the light microscope (BX51, Olympus, Tokyo, Japan). The evaluation was made by a single pathologist blindly without knowing the groups in the study. In the evaluation, semiquantitative scoring was performed according to the criteria used in an earlier study by Appleyard and Wallace (Table 2).^21^

### Statistical Analysis

All statistical evaluation of our study was performed using GraphPad Prism (version 7.04, GraphPad Software Inc., San Diego, Calif, USA) program. One-way analysis of variance was used for statistical evaluation between groups. In the interpretation of the values and the multiple comparisons of the group values, “Tukey’s multiple comparisons,” “Dunnett’s multiple-comparisons,” and “Sidak’s multiple comparisons” tests were applied. The values of the groups were expressed as standard deviations ± mean, and *P* < .05 was considered statistically significant. Excel (Microsoft, Wash, USA) program was used to prepare the graph showing the weight change and to determine the average values. The distributions of biochemical measurements were evaluated by the Shapiro–Wilk’s test and skewness/kurtosis statistics.

## Results

**Figure 1.**
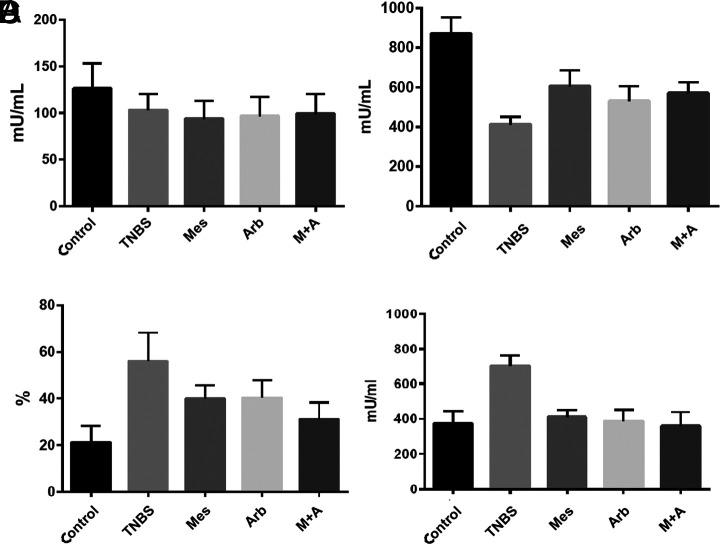
(A) Distribution of catalase values in study groups, (B) distribution of glutathione peroxidase values in study groups, (C) distribution of superoxide dismutase inhibition inhibition values in study groups, and (D) distribution of myeloperoxidase values in study groups. Arb, arbutin; Mes, mesalazine; TNBS, trinitrobenzene sulfonic acid.

Catalase level results showed the lowest values in the colitis group, although there was no statistically significant difference between the control group and the colitis and treatment groups (*P* > .05) (Figure 1A).

Increase has been observed in colon GPx levels in all treatment groups compared to the TNBS group (*P* < .05). We found an increase in GPx level (*P* < .005) compared to the TNBS group in the group that received mesalazine alone, a significant increase in GPx level compared to the TNBS group in the group that underwent mesalazine+arbutin (*P* < .01), and increase in the GPx level compared to the TNBS group in the group that received arbutin-alone (*P* < .05) (Figure 1B).

We found a statistically significant decrease in SOD inhibition in all treatment groups compared to the TNBS group (*P* < .05). This difference was significant in the group applied together with arbutin + mesalazine (*P* < .005) (Figure 1C).

Colon MPO levels were found to be highest in the TNBS group (*P* < .001). Both treatments applied caused a significant reduction, but there was no statistical difference between them (*P* > .05) (Figure 1D).

**Figure 2.**
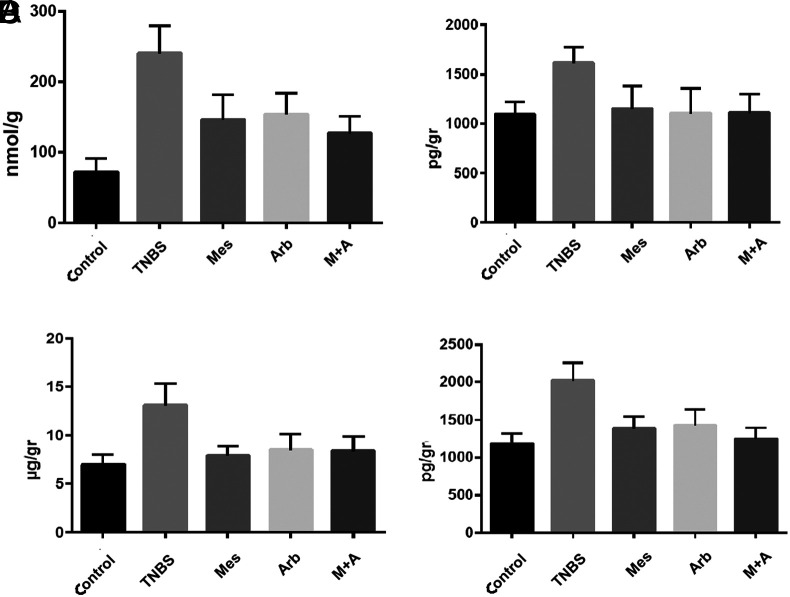
(A) Distribution of malondialdehyde values in working groups, (B) distribution of interleukin (IL)-1 β values in working groups, (C) distribution of IL-6 values in working groups, (D) distribution of tumor necrosis factor alpha values in study groups. Arb, arbutin; Mes, mesalazine; TNBS, trinitrobenzene sulfonic acid.

We found a decrease in MDA in all treatment groups compared to the TNBS group (*P* < .005). This significance is highest in arbutin + mesalazine group (*P* < .001) (Figure 2A).

We found a statistically significant decrease in tissue IL-1β levels in treatment groups against TNBS group (*P* < .01). When the treatment groups were compared among themselves, there was no significant difference in IL-1β levels between the treatment groups (*P* > .05) (Figure 2B).

There was a statistically significant decrease in tissue IL-6 levels in all treatment groups compared to the TNBS group (*P* < .005). This difference was more significant in the group that received mesalazine alone (*P* < .001) (Figure 2C).

The decrease in tissue TNF-α was statistically significant in all treatment groups compared to the TNBS group (*P* < .005). This difference was significant in the groups treated together with mesalazine and arbutin + mesalazine alone (*P* < .001) (Figure 2D).

All biochemical results are given in Table 3.

**Figure 3.**
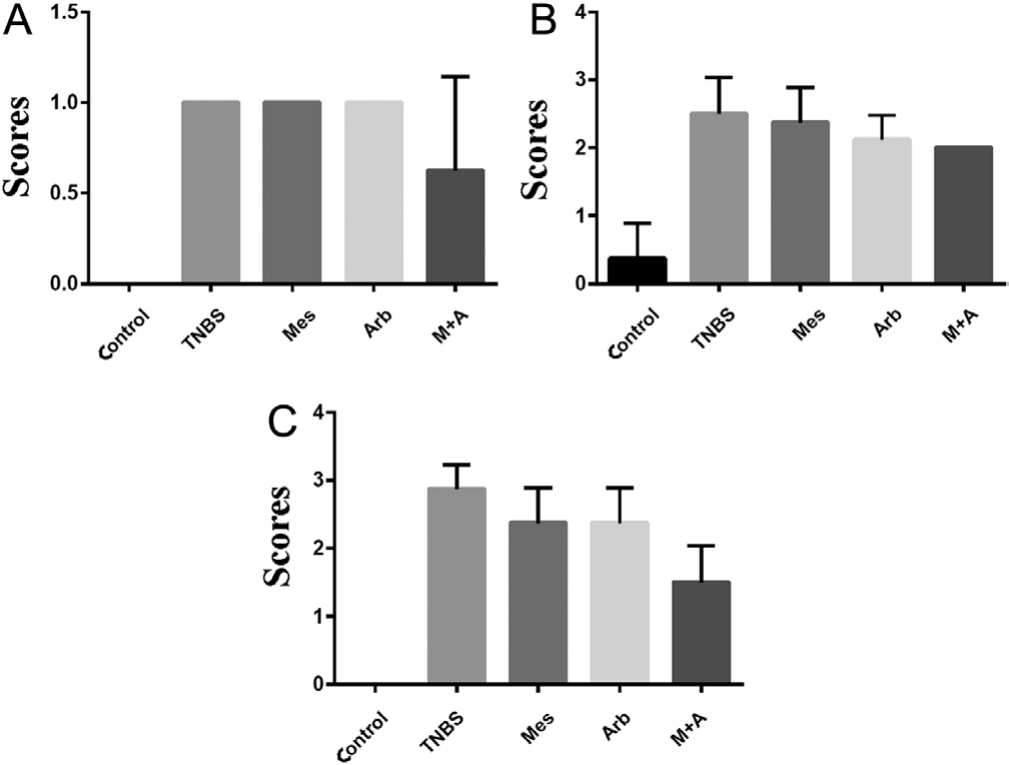
(A) Distribution of goblet cell reduction values in study groups. (B) Distribution of cell infiltration values in study groups. (C) Distribution of mucosal loss values in study groups.

**Figure 4.**
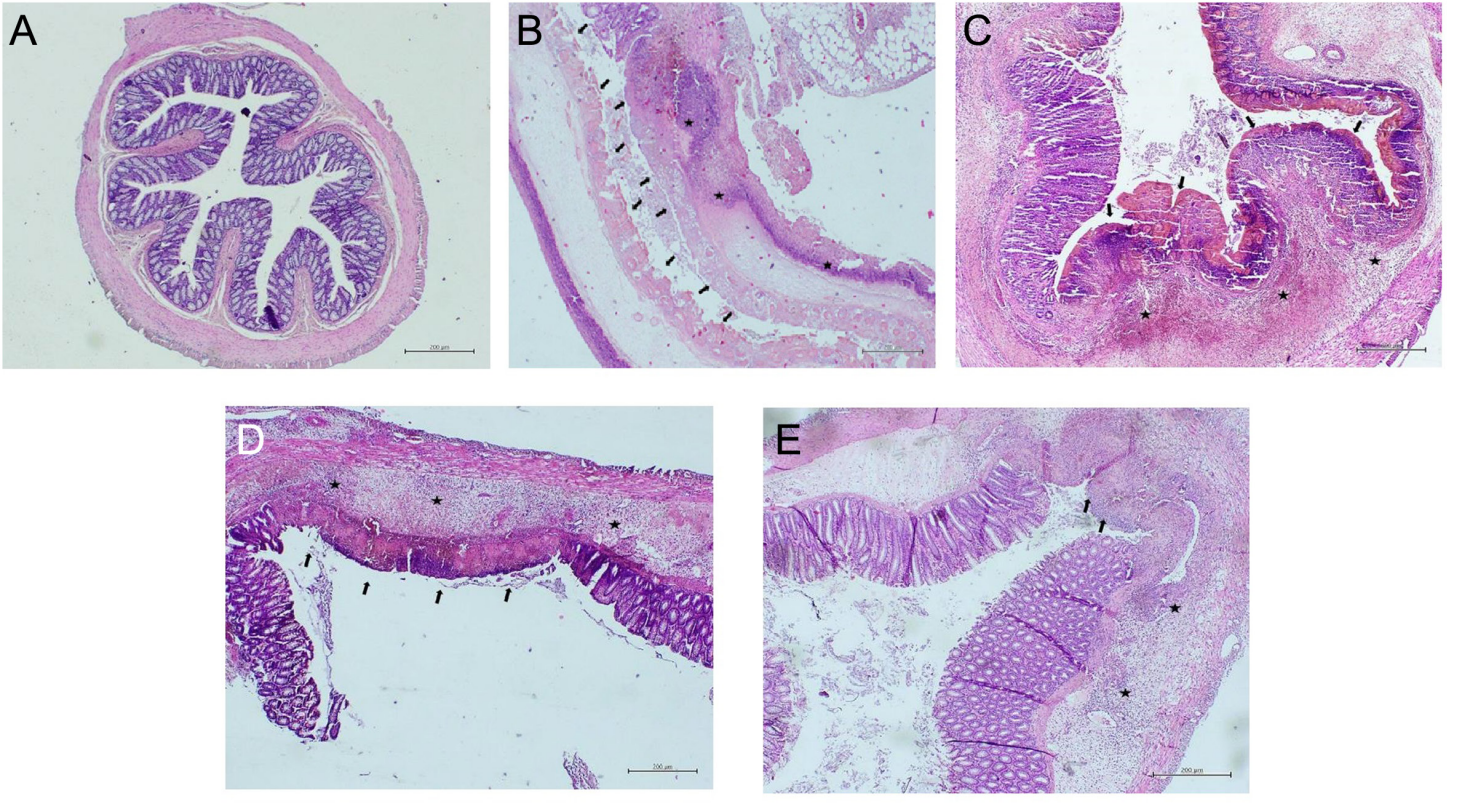
(A) Column with a normal appearance in the control group (HE,  ×40). (B) Colon with extensive ulceration (above 10%), lyricin cellular infiltration, and Goblet cell reduction in the TNBS group (HE, ×40). (C) Ulceration (5%-10%) in the mesalazine-treated group, colon with moderate cellular infiltration (HE, ×40). (D) Ulceration (5%-10%) in the Arbutin-treated group, colon with moderate cellular infiltration (HE, ×40). (E) Ulceration (less than 5%) in the group treated with mesalazine and arbutin, colon with minimal cellular infiltration (HE, ×40).

In histopathological tissue analysis, no statistically significant decrease in goblet cell reduction in the treatment groups against the TNBS group (*P* > .05) has been detected. Although there was an observational difference in the group in which mesalazine and arbutin were used together, there was no statistically significant difference (*P* > .05) (Figure 3A). Although cellular infiltration values were observed in histopathological evaluation compared to the TNBS group in the treatment groups, there was no significance (*P* > .05) (Figure 3B). In the histopathological evaluation of mucosal losses, although there was an observational difference in the arbutin and mesalazine groups alone compared to the TNBS group, there was no statistically significant decrease (*P* > .05). In the group where mesalazine and arbutin were used together, we found a statistically significant decrease in the mucosal loss value compared to the TNBS group (*P* < .05) (Figure 3C). Histopathological microscopic images are given in Figure 4.

All histopathological results are given in Table 4.

## Discussion

In our study, we investigated the possible positive effects of arbutin in the TNBS-induced experimental colitis model. We compared it with mesalazine used in the treatment of IBD and observed the effect of using it together. Proinflammatory cytokines (IL-6, IL-1β, TNF-α) and oxidant/antioxidant parameters (MDA, MPO, SOD inhibition, CAT, GPx) were processed in the study groups. In histopathological evaluation, goblet cell reduction, cellular infiltration and mucosal loss were evaluated. When the treatment groups were compared with the TNBS group, statistical significance was achieved in MDA, MPO, SOD inhibition, GPx values, IL-6, IL-1β, and TNF-α levels. In histopathological evaluation, a statistically significant decrease in mucosal loss was observed in the group where mesalazine and arbutin were used together compared to the TNBS group.

In our study, mesalazine, arbutin and mesalazine + arbutin were given 6 hours after TNBS application. Pontell et al^22^ in their study, showed that inflammation began 3 hours after TNBS injection into the ileal lumen and that the immediate damage caused by TNBS in the mucosa was rapidly reversed (<1 day).

In our we study found a statistically significant decrease in tissue MDA value in all treatment groups compared to the TNBS group. In the in vitro study of Takebayashi et al^16^ with arbutin, they reported that arbutin exerts antioxidant activity comparable to, or even more potent, than hydroquinone. In the gentamicin-induced experimental nephrotoxicity study conducted by Emadi et al,^23^ it was reported that the administration of arbutin prevented gentamicin-induced nephrotoxicity, and microscopic, oxidant, and antioxidant parameters supported the results. In our study, arbutin’s antioxidant efficacy was determined by biochemical and histopathological analyses. In the Alzheimer’s model conducted by Dastan et al,^24^ MDA levels decreased in the group that used arbutin as a preservative.

In our study, although tissue CAT levels were higher in the control group compared to colitis and treatment groups, there was no statistically significant difference. In a study conducted by Seyfizadeh et al^25^ with arbutin in Hep-G2 cells, it was observed that the administration of arbutin increased CAT and SOD levels and decreased MDA levels. In an experimental ethanol-induced liver injury model study conducted by Wang et al,^26^ a significant increase in SOD and GPx levels was found in rats treated with arbutin, and it was also shown that the levels of the inflammatory cytokines TNF-α and IL-6 decreased significantly in rats treated with arbutin.

We found a statistically significant decrease in MDA value in all treatment groups compared to the TNBS group. As for the SOD inhibition value, there was a statistically significant decrease in all treatment groups compared to the TNBS group, consistent with the increase in SOD levels shown in the studies.

Our study found a statistically significant increase in tissue GPx levels in all treatment groups compared to the TNBS group and a statistically significant decrease in TNF-α, IL-6, and IL-1β levels in all treatment groups compared to the TNBS group. In the lipopolysaccharide-induced experimental lung injury model conducted by Ye et al^27^ in rats, it was observed that the level of SOD increased and the MDA level decreased in the rats treated with arbutin compared to the control group. The inflammatory cytokines TNF-α, IL-6, and IL-1β were reduced in arbutin-treated mice compared to the control group.

In Bian et al’s^28^ experimental model of lipopolysaccharide-induced sepsis pneumonia in mice, it was observed that MPO and MDA levels decreased in mice treated with arbutin and levels of the inflammatory cytokines TNF-α, IL-6, and IL-1β in mice treated with arbutin. These results support our study and show that oxidative damage markers and inflammatory parameters are reduced.

In our study, no therapeutic agent was given before the formation of experimental colitis, and arbutin administration of 250 mg/kg was performed intragastric 3 times, the first of which was 6 hours after the experimental colitis was formed. Although histopathological examination revealed observational differences in goblet cell reduction and cellular infiltration in the treatment groups compared to the TNBS group, there was no statistically significant difference. A statistically significant decrease in the mucosal loss value was found in the group using mesalazine and arbutin together compared to the TNBS group. In the experimental study in which the gastroprotective property of arbutin was examined by Taha et al,^29^ 30 and 60 mg/kg arbutin was administered orally for 14 days. This study observed decreased ulcer area, submucosal edema, and leukocyte infiltration in rats treated with arbutin. The MDA levels in the stomach tissue decreased in rats treated with arbutin. The study observed that TNF and IL-6 levels increased in ulcerated rats compared to the control group, while they did not increase in rats treated with arbutin.

Our study found that TNF-α, IL-6, and IL-1β levels decreased statistically significantly in all treatment groups compared to the TNBS group. These results show that the anti-inflammatory property of arbutin is effective on the intestinal mucosa. In our study, it is essential that the anti-inflammatory properties of arbutin as well as the antioxidant properties of arbutin, were shown in the intestinal tissue and that arbutin and mesalazine were administered together and compared with other groups. Unlike other studies, the fact that we have administered arbutin in 3 repeated doses instead of in the form of low doses for a long time in the dose of 250 mg/kg may also be a guide for the use in the acute treatment of IBD exacerbations. In a dextran sodium sulfate (DSS)-induced experimental colitis model study conducted by Zhang et al^30^ in mice, intragastric arbutin was administered in doses of 50 mg/kg and 100 mg/kg for 7 days to mice with experimental colitis. In histopathological examination, The total histological colitis score was calculated by scoring the severity, degree of inflammation, crypt damage, and participation in percentage. Histopathological improvement was detected in the groups treated with arbutin. Wang et al^31^ conducted a DSS-induced experimental colitis model study in mice that evaluated the anti-inflammatory properties of arbutin. In the study, intragastric arbutin was administered to mice with experimental colitis for 7 days at doses of 50 mg/kg and 100 mg/kg. The results were evaluated by comparing them with sulfasalazine. Histopathological examination evaluated goblet cell loss, crypt loss, epithelial damage, and mucosal infiltration and scored. In the group treated with arbutin, histopathological examination showed significant improvement. In our study, unlike these 2 studies, we created the experimental colitis model with TNBS. We used mesalazine instead of the sulfasalazine Wang et al^31^ used and compared it to arbutin. We also compared it with other groups by applying arbutin and mesalazine together. The total dose of arbutin administered in our study was similar to the maximum total dose of Wang et al.^31^ Although histopathological examination revealed observational differences in goblet cell reduction and cellular infiltration in the treatment groups compared to the TNBS group, there was no statistically significant difference. We found a statistically significant decrease in the mucosal loss value in the group using mesalazine and arbutin together compared to the TNBS group. The study by Wang et al^31^ calculated and evaluated the total histological score. Our study evaluated goblet cell reduction, cellular infiltration, and mucosal loss values separately. The difference in the results of the histopathological examination depends on this.

Although the model used in the study is important for the healing processes of the colon since it is based on TNBS damage, it does not carry all the pathophysiological details of the UC process. In our study, one of the limitations of the study is that sacrification was not performed at the sixth hour to confirm whether colitis occurred within 6 hours as a result of TNBS induction in rats and that the number of groups and animals in the groups was limited.

Although the use of rats of both genders in our experimental modeling would be of great importance in revealing the healing process and effect differences between the genders, it was determined that male rats/mice were especially preferred in experimental studies to be hormonally stable and to eliminate the effects of hormonal changes on the healing processes.^28,29,31^ Male rats were used in the study because the parameters examined in our study focused on the intergroup differences between the treated and untreated rats and the untreated rats regardless of gender. Only male rats were used in the study, and therefore, the inability to make comparisons between genders and the inability to molecularly examine the biochemical parameters examined at the transcription and translation level due to limited budget are limitations.

In conclusion, in our study, it was thought that arbutin had a protective antioxidant effect on SOD, MDA, and MPO in colitis-induced rats. Statistically significant decrease in TNF-α, IL-6, and IL-1β values in groups treated with arbutin shows the anti-inflammatory efficacy of arbutin. In our study, biochemical and histopathological results showed that arbutin administration revealed positive results in colitis-induced rats. The use of arbutin alone or in combination with mesalazine in UC is promising for the future in terms of treatment. We think further studies on this subject will help understand the therapeutic properties of arbutin on UC.

## Figures and Tables

**Table 1.** Experimental Diagram

	Hour 0	Hour 6	Hour 30	Hour 54	Hour 72
Sham control	Intrarectal saline	First saline gavage	Second saline gavage	Third saline gavage	Sacrification
TNBS	Colitis induced	–	–	–	Sacrification
Mes	Colitis induced	First Mes gavage	Second Mes gavage	Third Mes gavage	Sacrification
Arb	Colitis induced	First Arb gavage	Second Arb gavage	Third Arb gavage	Sacrification
Mes + arb	Colitis induced	First Mes + arb gavage	Second Mes + arb gavage	Third Mes + arb gavage	Sacrification

Arb, arbutin; Mes, mesalazine; TNBS, trinitrobenzene sulfonic acid.

**Table 2.** Scoring Criteria Used in the Pathological Microscopic Evaluation

Score	Loss of Mucous Membranes	Cellular Infiltration	Crypt Abscess	Goblet Cell Reduction (Necrosis/Ulceration)
0	No	No	No	No
1	Under 5%	Few	Apparent	Apparent
2	5%-10%	Middle	-	-
3	Over 10%	Apparent	-	-

**Table 3.** Biochemical Parameter Values

	Control	TNBS	Mes	Arb	M + A
MPO (mU/mL)	373 000 ± 71 621	701 000 ± 61 031	410 333 ± 39 807	385 833 ± 66 406	357 167 ± 81 263
CAT (mU/mL)	126 167 ± 27 088	102 833 ± 17 394	93 666 ± 19 551	96 666 ± 20 412	99 000 ± 21 410
GPx (mU/mL)	870 667 ± 83 377	412 167 ± 38 602	605 000 ± 81 545	530 167 ± 75 358	570 667 ± 55 164
IL-1β (pg/g)	1092 670 ± 129 728	1613 670 ± 163 867	1152 170 ± 226 414	1102 500 ± 256 475	1109 000 ± 186 654
IL-6 (pg/g)	6950 ± 1059	13 083 ± 2235	7866 ± 1032	8500 ± 1663	8383 ± 1479
MDA (nmol/g)	71 833 ± 19 446	240 333 ± 39 195	146 167 ± 35 391	153 833 ± 30 393	127 000 ± 24 025
SOD inhibition (%)	21 166 ± 7082	56 000 ± 12 280	39 833 ± 5845	40 333 ± 7501	31 000 ± 7375
TNF-α (pg/g)	1179 330 ± 140 773	2014 650 ± 242 701	1386 670 ± 157 504	1425 920 ± 219 728	1245 830 ± 148 817

Arb, arbutin; CAT, catalase; GPx, glutathione peroxidase; IL, interleukin; MDA, malondialdehyde; Mes, mesalazine; MPO, myeloperoxidase; SOD, superoxide dismutase inhibition; TNBS, trinitrobenzene sulfonic acid; TNF-α, tumor necrosis factor-alpha.

**Table 4.** Histological Parameter Values

	Control	TNBS	Mes	Arb	M + A
Goblet cell reduction	0.000 ± 0.000	1.000 ± 0.000	1.000 ± 0.000	1.000 ± 0.000	0.625 ± 0.517
Cellular infiltration	0.375 ± 0.517	2.500 ± 0.534	2.375 ± 0.517	2.125 ± 0.353	2.000 ± 0,000
Mucosal loss	0.000 ± 0.000	2.875 ± 0.353	2.375 ± 0.517	2.375 ± 0.517	1.500 ± 0.534

Arb, arbutin; Mes, mesalazine; TNBS, trinitrobenzene sulfonic acid.

## References

[1] Guan Q . A comprehensive review and update on the pathogenesis of inflammatory bowel disease. J Immunol Res. 2019;2019:7247238. (10.1155/2019/7247238) 31886308 PMC6914932

[2] Monteleone G Fina D Caruso R Pallone F . New mediators of immunity and inflammation in inflammatory bowel disease. Curr Opin Gastroenterol. 2006;22(4):361 364. (10.1097/01.mog.0000231808.10773.8e) 16760750

[3] Kornbluth A Sachar DB . Gastroenterology PPCotACo. Ulcerative Colitis Practice Guidelines in Adults: American College of Gastroenterology, Practice Parameters Committee. 2010;105(3):501 523. 10.1038/ajg.2009.727 20068560

[4] Silva I Pinto R Mateus V . Preclinical study in vivo for new pharmacological approaches in inflammatory bowel disease: a systematic review of chronic model of TNBS-induced colitis. J Clin Med. 2019;8(10):1574. (10.3390/jcm8101574) 31581545 PMC6832474

[5] Goyette P Labbé C Trinh TT Xavier RJ Rioux JD . Molecular pathogenesis of inflammatory bowel disease: genotypes, phenotypes and personalized medicine. Ann Med. 2007;39(3):177 199. (10.1080/07853890701197615) 17457716

[6] Sedghi S Fields JZ Klamut M , et al. Increased production of luminol enhanced chemiluminescence by the inflamed colonic mucosa in patients with ulcerative colitis. Gut. 1993;34(9):1191 1197. (10.1136/gut.34.9.1191) 8406152 PMC1375452

[7] Williams JG Hughes LE Hallett MB . Toxic oxygen metabolite production by circulating phagocytic cells in inflammatory bowel disease. Gut. 1990;31(2):187 193. (10.1136/gut.31.2.187) 2311976 PMC1378378

[8] Wang Y Parker CE Bhanji T Feagan BG MacDonald JK . Oral 5-aminosalicylic acid for induction of remission in ulcerative colitis. CDSR. Cochrane Database Syst Rev. 2016;2016(4):CD000543. (10.1002/14651858.CD000543.pub4) PMC7045743 27101467

[9] Feagan BG MacDonald JK . Oral 5-aminosalicylic acid for maintenance of remission in ulcerative colitis. CDSR. Cochrane Database Syst Rev. 2012;10:CD000544. (10.1002/14651858.CD000544.pub3) 23076890

[10] Carmen P Vlase L Tamas M . Natural resources containing arbutin. Determination of arbutin in the leaves of Bergenia crassifolia (L.) Fritsch. acclimated in Romania. Not Bot Horti Agrobot. 2009;37(1):129 132.

[11] Lindpaintner E . Arbutin und Methylarbutin und ihre Bestimmung in Drogen. Mit Unterstützung der Deutschen Forschungsgemeinschaft. Arc Pharm. 1939;277(9):398 415. (10.1002/ardp.19392770903)

[12] Yamaha T Cardini CE . The biosynthesis of plant glycosides. I. Monoglucosides. Arch Biochem Biophys. 1960;86(1):127 132. (10.1016/0003-9861(60)90379-9) 13846402

[13] Garrett JT . The Cherokee Herbal: Native Plant Medicine from the Four Directions. Simon and Schuster; 2003.

[14] Garcia-Jimenez A Teruel-Puche JA Berna J Rodriguez-Lopez JN Tudela J Garcia-Canovas F . Action of tyrosinase on alpha and beta-arbutin: a kinetic study. PLoS One. 2017;12(5):e0177330. (10.1371/journal.pone.0177330) 28493937 PMC5426667

[15] Xu WH Liang Q Zhang YJ Zhao P . Naturally occurring arbutin derivatives and their bioactivities. Chem Biodivers. 2015;12(1):54 81. (10.1002/cbdv.201300269) 25641837

[16] Takebayashi J Ishii R Chen J Matsumoto T Ishimi Y Tai A . Reassessment of antioxidant activity of arbutin: multifaceted evaluation using five antioxidant assay systems. Free Radic Res. 2010;44(4):473 478. (10.3109/10715761003610760) 20166881

[17] Pečivová J Nosál’ R Sviteková K Mačičková T . Arbutin and decrease of potentially toxic substances generated in human blood neutrophils. Interdiscip Toxicol. 2014;7(4):195 200. (10.2478/intox-2014-0028) 26109900 PMC4436208

[18] Zhou W Chen K Lu Q , et al. The protective effect of rosavin from Rhodiola rosea on radiation-induced intestinal injury. Chem Biodivers. 2020;17(12):e2000652. (10.1002/cbdv.202000652) 33089958

[19] Ek RO Serter M Ergin K , et al. The effects of caffeic acid phenethyl ester (CAPE) on TNBS-induced colitis in ovariectomized rats. Dig Dis Sci. 2008;53(6):1609 1617. (10.1007/s10620-007-0056-2) 17957471

[20] Deniz M Çetinel S Kurtel H . Blood flow alterations in TNBS-induced colitis: role of endothelin receptors. Inflamm Res. 2004;53(7):329 336. (10.1007/s00011-004-1266-0) 15241569

[21] Appleyard CB Wallace JL . Reactivation of hapten-induced colitis and its prevention by anti- inflammatory drugs. Am J Physiol. 1995;269(1):G119 G125. (10.1152/ajpgi.1995.269.1.G119) 7631788

[22] Pontell L Castelucci P Bagyánszki M , et al. Structural changes in the epithelium of the small intestine and immune cell infiltration of enteric ganglia following acute mucosal damage and local inflammation. Virchows Arch. 2009;455(1):55 65. (10.1007/s00428-009-0795-x) 19517133

[23] Emadi E Pouramir M Ghasemi-Kasman M Feizi F Halalkhor S Moghadamnia AA . Arbutin attenuates nephrotoxicity induced by gentamicin. Avicenna J Phytomed. 2021;11(3):210 217. 34046317 PMC8140210

[24] Dastan Z Pouramir M Ghasemi-Kasman M , et al. Arbutin reduces cognitive deficit and oxidative stress in animal model of Alzheimer’s disease. Int J Neurosci. 2019;129(11):1145 1153. (10.1080/00207454.2019.1638376) 31251091

[25] Seyfizadeh N Tazehkand MQ Palideh A , et al. Is arbutin an effective antioxidant for the discount of oxidative and nitrosative stress in Hep-G2 cells exposed to tert-butyl hydroperoxide? Bratisl Lek Listy. 2019;120(8):569 575. (10.4149/BLL_2019_093) 31379179

[26] Wang R Mu J . Arbutin attenuates ethanol-induced acute hepatic injury by the modulation of oxidative stress and Nrf-2/HO-1 signaling pathway. J Biochem Mol Toxicol. 2021;35(10):e22872. (10.1002/jbt.22872) 34346143

[27] Ye J Guan M Lu Y Zhang D Li C Zhou C . Arbutin attenuates LPS-induced lung injury via Sirt1/Nrf2/NF-κBp65 pathway. Pulm Pharmacol Ther. 2019;54:53 59. (10.1016/j.pupt.2018.12.001) 30528955

[28] Bian XX Zhao X Ma CH Shen CP . Arbutin alleviates LPS induced sepsis pneumonia in mice. Evid Based Complement Alternat Med. 2022;2022:5863952. (10.1155/2022/5863952) 35469161 PMC9034914

[29] Taha MME Salga MS Ali HM Abdulla MA Abdelwahab SI Hadi AHA . Gastroprotective activities of Turnera diffusa Willd. ex Schult. revisited: role of arbutin. J Ethnopharmacol. 2012;141(1):273 281. (10.1016/j.jep.2012.02.030) 22374081

[30] Zhang C Zhu H Jie H Ding H Sun H . Arbutin ameliorated ulcerative colitis of mice induced by dextran sodium sulfate (DSS). Bioengineered. 2021;12(2):11707 11715. (10.1080/21655979.2021.2005746) 34783296 PMC8809946

[31] Wang L Feng Y Wang J , et al. Arbutin ameliorates murine colitis by inhibiting JAK2 signaling pathway. Front Pharmacol. 2021;12:683818. (10.3389/fphar.2021.683818) 34594215 PMC8477021

